# Refined Exclusion in Medical AI: Data Justice and Patient Safety Governance

**DOI:** 10.2196/102359

**Published:** 2026-10-01

**Authors:** Jae Hyun Lee, Boram Choi, Kwunho Jeong, Sang Won Suh, Ju Han Kim, Dae-Soon Son

**Affiliations:** 1 Global Research Center JNPMEDI Seoul Republic of Korea; 2 Department of Clinical Research Onu Institute Seoul Republic of Korea; 3 Department of Physiology College of Medicine Hallym University Chuncheon, Gangwon Republic of Korea; 4 Division of Biomedical Informatics, Seoul National University Biomedical Informatics (SNUBI) College of Medicine Seoul National University Seoul Republic of Korea; 5 Major in Bio-Healthcare Convergence College of Natural Sciences Hallym University Chuncheon, Gangwon Republic of Korea; 6 Institute of New Frontier Research, Division of Big Data and Artificial Intelligence College of Medicine Hallym University Chuncheon, Gangwon Republic of Korea; 7 Hallym AI-BioHealth R&BD Center Research Institute of Medical-Bio Convergence Hallym University Chuncheon, Gangwon Republic of Korea

**Keywords:** medical artificial intelligence, medical AI, algorithmic fairness, data justice, refined exclusion, health equity, patient safety, artificial intelligence governance, AI governance

## Abstract

Medical AI is often evaluated using aggregate measures of discrimination, calibration, and accuracy. However, these measures can obscure clinically important variation across patient groups, institutions, devices, and workflows. This viewpoint defines refined exclusion as a governance condition in which an AI system appears successful in aggregate, while uncertainty, error, or reduced clinical reliability is concentrated in populations that are insufficiently represented, measured, validated, or monitored. The concept does not replace algorithmic fairness, hidden stratification, dataset shift, or subgroup performance analysis. It connects these mechanisms to a distinct consequence: an unequal distribution of safety that remains inadequately detected or corrected. Drawing on purposively selected, illustrative evidence from population health management, chest radiography, dermatology, computational pathology, medical foundation models, and clinical measurement, we distinguish model-level disparity, patient safety signals, and documented patient harm. We then frame data justice as a complementary governance approach with distributional, procedural, and substantive dimensions. The proposed lifecycle decision gates address intended use, subgroup learnability, data provenance, validation, procurement, local deployment, monitoring, updates, and patient feedback. Each gate links minimum evidence to decision authority and 1 of 4 actions: proceed, enrich or validate, restrict use, or pause or retire. Governance intensity should be proportionate to clinical risk and evidentiary uncertainty. By linking subgroup evidence gaps to institutional decisions and corrective action, the framework shifts attention from whether a model performs well on average to whether its safety is demonstrable for the populations and settings in which it will be used.

## Introduction

AI is increasingly used in medical imaging, electronic health record prediction, digital pathology, population health management, and clinical communication tools. Many systems achieve strong performance on narrowly defined tasks, while foundation models are expanding the range of tasks a single model may support [1,2]. However, performance averaged across a development or validation cohort does not establish equal reliability across the patient groups, institutions, devices, and workflows encountered in practice [3-5].

These concerns have generated substantial work on algorithmic fairness, data quality, reporting, and trustworthy AI. Contemporary fairness research examines not only output disparities but also data acquisition, labeling, measurement, causal structure, and deployment context [3,5]. International guidance similarly emphasizes equity, transparency, robustness, traceability, and lifecycle governance [6-8]. The remaining gap is not simply a lack of metrics or principles. Rather, it is the absence of an explicit bridge from subgroup evidence to institutional decision-making, including corrective authority when clinical reliability is distributed unevenly.

We use the term refined exclusion for this governance problem. It describes a condition in which an AI system maintains acceptable aggregate performance while uncertainty, error burden, or reduced reliability is concentrated in populations insufficiently characterized within the data ecosystem, and governance processes do not adequately detect, communicate, or correct that concentration. The exclusion is “refined” because it need not involve overt denial or intentional discrimination. It may arise through apparently neutral choices about targets, labels, subgroup definitions, validation cohorts, thresholds, or monitoring.

Refined exclusion addresses a dimension of governance distinct from lifecycle governance in adaptive medical AI. Co–Lifecycle Governance emphasizes maintaining alignment among validation, change management, surveillance, and accountability as a model evolves over time [9]. On the other hand, refined exclusion focuses on whose safety remains visible throughout the lifecycle, particularly when evidence is sparse or subgroup-specific performance is obscured by aggregate evaluation. The frameworks intersect when an update improves overall performance while degrading reliability for a smaller or less visible population.

The primary audience for this viewpoint is health system decision-makers responsible for evaluating, procuring, validating, deploying, and overseeing medical AI, including clinical informatics teams and institutional AI governance or patient safety committees. Developers, vendors, regulators, and researchers constitute a secondary audience, as implementation requires shared evidence and accountability across organizations.

### Approach and Scope

This is a conceptual viewpoint, not a systematic or scoping review. Cases were purposively selected based on whether they provided peer-reviewed empirical evidence of subgroup disparity or proxy or measurement failure, represented a distinct data modality or lifecycle stage, and had direct relevance to clinical decisions. No formal database search, screening flow, or risk-of-bias assessment was performed. The cases are intended to illustrate recurring mechanisms rather than estimate the prevalence of inequity. They provide the basis for defining the concept, establishing its conceptual boundaries, and developing an operational framework for future empirical evaluation.

### Defining Refined Exclusion

Refined exclusion is present when 4 conditions coexist. First, a clinically relevant population or context is insufficiently represented, measured, labeled, validated, or monitored. Here, “data-poor” does not mean only a small number of records; it also includes incomplete labels, unreliable measurements, sparse outcomes, weak institutional coverage, or insufficient evidence for the intended setting. Second, the resulting vulnerability is obscured by aggregate evaluation or by subgroup categories that are too broad to reveal it. Third, uncertainty, misclassification, reduced utility, or workflow burden becomes concentrated in the affected population or context. Fourth, governance arrangements fail to convert that signal into an appropriate decision, such as additional validation, restricted use, workflow safeguards, or suspension.

Ordinary model error affects performance; refined exclusion affects the distribution of safety. This distinction does not imply that every subgroup difference constitutes exclusion. Random variation, multiple testing, differences in disease prevalence, and small samples can produce unstable estimates. Refined exclusion instead requires a clinically meaningful pattern, a plausible pathway from the data or workflow to concentrated risk, and inadequate recognition or response by the organizations responsible for the system.

Refined exclusion is related to, but not synonymous with, several established concepts.

Hidden stratification describes a technical failure in which clinically meaningful subsets are obscured within broad labels or tasks [10]. Dataset shift describes changes between development and deployment distributions that can impair generalization [11]. Subgroup underperformance is an observed difference in discrimination, calibration, error, or utility. Algorithmic fairness is the broader field that identifies and mitigates unjustified disparities across data, models, and decisions [3,12]. Health inequity is a broader concept that encompasses unequal opportunities and outcomes arising from social and institutional structures. These concepts can identify mechanisms, signals, or contexts. Refined exclusion names the governance condition that arises when such signals are hidden by aggregate success and are not adequately controlled (Table 1).

**Table 1:** Conceptual boundaries of refined exclusion.

Construct	Primary focus	What it identifies	Relationship to refined exclusion
Algorithmic bias and fairness	Data, model behavior, decisions, and their social context	Unjustified disparities and methods for assessment or mitigation	Broad field that supplies evidence and methods; refined exclusion does not replace it
Hidden stratification	Unrecognized clinically meaningful subsets within a broad task or label	Subset-specific failure concealed by aggregate performance	A possible technical mechanism of refined exclusion
Dataset shift	Differences between development and deployment distributions	Loss or alteration of performance across settings or time	A possible trigger or amplifier of refined exclusion
Subgroup underperformance	Performance within a prespecified group	Differences in discrimination, calibration, error rates, or utility	A potential safety signal; not sufficient by itself to establish refined exclusion
Health inequity	Unequal health opportunities, care, or outcomes	Structural disparities within populations and institutions	The broader context in which refined exclusion may arise or have consequences
Refined exclusion	The data-to-deployment governance system	Aggregate success coexisting with concentrated uncertainty or reduced reliability and inadequate remediation	A governance condition concerning the unequal distribution of safety

### Representative Mechanisms Across Medical AI

The population health management example reported by Obermeyer et al [13] illustrates proxy target selection. A widely used algorithm predicted health care cost as a proxy for health need. Because Black patients with comparable levels of illness incurred lower health care spending, the algorithm estimated their risk as lower and consequently reduced their eligibility for additional care management. This represented target misspecification arising from a structurally patterned proxy rather than conventional target leakage. It produced a documented allocation consequence and shows why a prediction target must be clinically justified, not merely convenient or predictive.

Medical imaging shows how aggregate performance can conceal subgroup-specific error. Chest radiograph classifiers have demonstrated underdiagnosis disparities across demographic and intersectional groups [14]. The observed disparity does not establish a single cause; prevalence, labels, acquisition practices, thresholds, and site effects may contribute. Dermatology models evaluated on a pathologically confirmed, diverse image set performed less well for darker skin tones and uncommon diseases [15]. Computational pathology models have also shown demographic variation in misdiagnosis, with potential contributions from site, scanner, staining, tissue processing, tumor subtype, and label heterogeneity [16]. These studies establish model-level disparities and clinically plausible safety signals, but they do not consistently measure downstream clinical harm. Governance should therefore report subgroup estimates and uncertainty, investigate plausible mechanisms, and specify the clinical consequences of false-positive and false-negative errors without overstating the evidence.

Foundation models extend these concerns across tasks. Vision-language models for chest radiography have demonstrated demographic differences in underdiagnosis, and evaluations of a general-purpose language model have identified race- and gender-associated variation in clinical outputs [17,18]. Mechanisms that can contribute to refined exclusion include opaque pretraining data, instruction tuning, default prompts, multimodal alignment, limited language coverage, and task-specific deployment. Unsupported reasoning may further amplify harm across prompts, languages, and clinical contexts, although subgroup-specific evidence for this pathway remains limited.

Validation of one benchmark or prompt configuration cannot establish safety across other tasks, populations, model versions, or workflows.

Upstream measurement can also transmit inequity into AI. Pulse oximetry has shown differential measurement error across racialized groups [19]. A biased measurement may become an AI input, training label, prediction target, or reference standard. An apparently well-performing model may therefore reproduce measurement error before any model-level fairness intervention is applied. Data provenance must include the reliability of the instruments and clinical processes that generated the data.

Generalization is a further cross-cutting mechanism. Subgroup reliability may also deteriorate when models move across institutions and populations, even when fairness appears acceptable in the development setting [20]. Table 2 distinguishes what each example demonstrates from the governance response it warrants.

**Table 2:** Illustrative mechanisms, evidence, and governance responses.

Domain	Mechanism	Measurable signal	Evidence status	Governance response
Population health management	Cost or use used as a proxy for clinical need	Risk score and care allocation by group at comparable levels of illness burden	Documented allocation consequence	Reassess target validity, replace or constrain proxy, and re-evaluate eligibility decisions
Chest radiograph AI	Label, prevalence, threshold, or generalization differences	Sensitivity, false-negative rate, calibration, and CIs by demographic and intersectional group	Model disparity and potential safety signal	External and local validation, threshold review, workflow safeguard, and postdeployment monitoring
Dermatology AI	Limited representation across skin tones and disease spectra	Discrimination and error by skin tone and disease frequency	Model disparity	Document skin tone and disease coverage, enrich data, and restrict unsupported uses
Computational pathology	Demographic and institutional heterogeneity in slides and labels	Misdiagnosis and error patterns by demographic group and site	Model disparity	Multisite evaluation; provenance for scanner, stain, site, and labels; and investigate failure clusters
Foundation models	Pretraining, instruction, prompt, alignment, and task-context effects	Output differences across demographic attributes, prompts, languages, and clinical tasks	Model disparity; downstream harm generally not established	Task- and prompt-specific evaluation, version control; guardrails, and user feedback and incident review
Measurement-stage inequity	Differential device or measurement error	Residual error and missed abnormality across racialized groups or other clinically relevant strata	Upstream measurement disparity	Audit measurement provenance, avoid biased labels or reference standards, and validate alternative measurements

### From Performance Disparity to Patient Safety Signal

Patient safety claims require evidentiary discipline. We distinguish 3 levels. A model-level disparity is a difference in performance or error across groups. A patient safety signal is a disparity or pattern that, given the intended use and workflow, could plausibly contribute to preventable harm and therefore requires investigation or control. Documented patient harm requires evidence of an actual downstream consequence, such as delayed diagnosis, inappropriate triage, treatment omission, or exclusion from beneficial care. Progressing from the first to the third level requires evidence of effects on clinical outcomes and workflows, rather than rhetoric alone. No universal numerical threshold can determine when a disparity becomes a safety signal. Trigger criteria should be prespecified according to intended use, severity of potential harm, the relative importance of false-negative and false-positive errors, subgroup calibration, decision thresholds, clinical utility or net benefit, and statistical uncertainty [4,21]. A high-risk diagnostic system may justify a more stringent prespecified tolerance for subgroup performance differences and earlier escalation, whereas a low-risk administrative tool may warrant lighter monitoring. When subgroup sample sizes are too small for stable estimates, the absence of statistical significance should not be treated as evidence of safety. Wide CIs and missing evidence should be recorded as uncertainty and linked to proportionate safeguards. At minimum, formal review should be triggered by a serious AI-associated incident; a breach of a prespecified subgroup performance or calibration criterion; or a recurrent cluster of concordant errors, delays, or complaints.

### Data Justice as a Complementary Governance Framework

Data justice provides a useful bridge from fairness evidence to institutional action. In its broader formulation, data justice examines how data systems determine whose experiences are made visible, how people are represented, and how they are treated [22]. Applied to medical AI, it asks whether data practices and governance arrangements make safety demonstrable for the populations and settings within the intended use. This framing complements algorithmic fairness rather than superseding it: fairness methods identify disparities and possible mitigations, while data justice assigns significance to representation, process, and real-world consequence within a clinical governance structure.

Distributional justice concerns who and what are represented. Relevant dimensions are task-specific and may include age, sex, race and ethnicity, skin tone, disability, language, socioeconomic circumstances, geography, disease severity, comorbidities, institution, device, and workflow. These categories are not interchangeable, and demographic categories should not be used as crude biological proxies. The aim is to identify variables linked to plausible failure mechanisms. Intersectional analysis is important when risk may emerge only from combinations of characteristics, but it should be planned with attention to sample size, privacy, and multiplicity.

Procedural justice concerns how data and decisions are produced. It requires documentation of data provenance, measurement processes, inclusion and exclusion criteria, label generation and uncertainty, missingness, proxy selection, preprocessing, intended use boundaries, and model versions. Datasheets, model cards, and reporting standards, such as TRIPOD+AI (Transparent Reporting of a Multivariable Prediction Model for Individual Prognosis or Diagnosis–AI), provide valuable components [23-25]. Data justice adds a governance requirement: disclosed limitations must be connected to a responsible reviewer, an institutional decision, and a plan for monitoring or correction.

Substantive justice focuses on the consequences of how systems are used in practice. It examines whether deployment changes error patterns, access to care, diagnostic timing, clinician workload, or patients’ experiences. Relevant evidence may include subgroup calibration drift, false-negative burden, alert and override patterns, referral or allocation differences, incidents, and patient complaints. The 3 dimensions are interdependent: representation without procedural traceability can be superficial, documentation without decision authority can become paperwork, and outcome monitoring without upstream evidence may detect harm too late. Figure 1 summarizes the 4 defining conditions of refined exclusion and shows how data justice connects subgroup safety signals to accountable governance actions.

**Figure 1:**
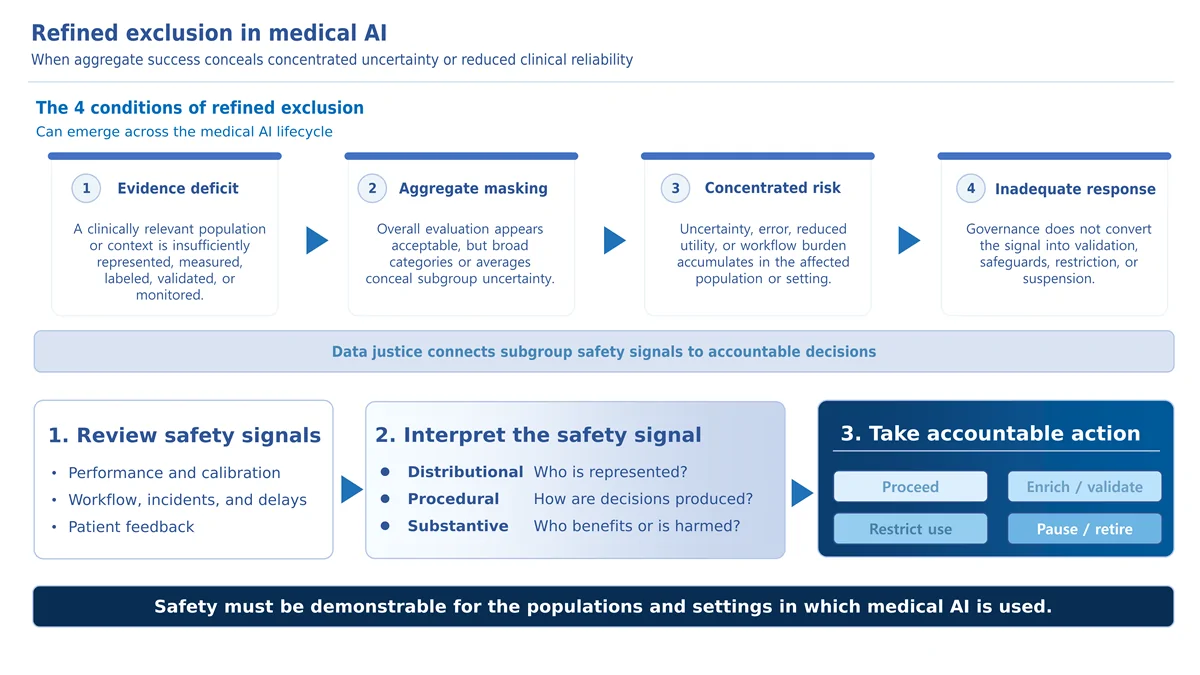
From refined exclusion to accountable governance action.

### Operationalizing Data Justice Across the Medical AI Lifecycle

Operationalization requires decision gates rather than an open-ended checklist. At each stage, a health system should specify minimum evidence, the reviewer, the authority empowered to act, and the available decision. We propose 4 actions: proceed, enrich data or validate further, restrict use or add safeguards, and pause or retire. Decisions should be documented and revisited when the model, population, workflow, or evidence changes.

At the intended use and development stages, developers and clinical partners should define the clinical task, target population, excluded uses, decision threshold, and consequences of error. They should assess subgroup learnability, that is, whether available samples, events, labels, measurements, and site coverage are sufficient to estimate clinically relevant patterns for the proposed use [26]. Practical indicators include subgroup sample size and event count, label completeness and uncertainty, missingness, representation across sites and devices, discrimination, calibration, CI width, feature stability, and the feasibility of intersectional analysis. Failure to establish learnability should lead to data enrichment, narrower intended use, explicit uncertainty warnings, external validation, or nondeployment rather than a generic limitation statement.

At validation and procurement, overall discrimination is insufficient. Evaluation should include calibration, threshold-specific error, and clinical utility when applicable, as discrimination may remain acceptable while risks are poorly calibrated or decisions are unfavorable for a subgroup [21,25]. Vendors should provide an evidence dossier covering training and validation populations, data and label provenance, subgroup analyses, known failure modes, regulatory status, updates, version control, local validation support, data access and sharing terms necessary for local validation and postdeployment audit, and contractual duties for monitoring and incident response. Committee composition should be proportionate to risk and include relevant clinical, informatics, safety, legal, and patient perspectives.

Local validation should test the model in the population, devices, and workflow in which it will be used. The institution should map where the output enters the care pathway, who may override it, what happens when the model is unavailable, and how errors can be detected. Early clinical evaluation and medical algorithmic audit frameworks provide useful foundations for examining human-AI interaction and error pathways [27,28]. Where local sample sizes are insufficient, institutions may use external multisite evidence, staged deployment, enhanced human review, or restricted use while collecting prospective data. They should not convert an evidence gap into a claim of equivalence.

Postdeployment monitoring should combine model and workflow indicators, including subgroup discrimination and calibration, threshold-specific errors, alerts, overrides, diagnostic delay, referral or care allocation, downtime, complaints, and incidents, as relevant. Monitoring should be signal-oriented, that is, causality need not be established before review begins. A severe event or repeated pattern should trigger documented triage; case review; subgroup revalidation; workflow analysis; and a decision to continue, recalibrate, restrict, pause, or retire the system [27,29].

Patient participation should be integrated as safety intelligence rather than treated as symbolic consultation. Institutions should provide a visible channel for patients and caregivers to report inaccurate characterization, inaccessible language, repeated routing errors, or other AI-associated concerns. A complaint should be linked to the model version, clinical context, and available subgroup information. A severe complaint, or a cluster involving a common population or workflow, should trigger formal review by the designated AI governance or patient safety body. Patient perspectives may also inform which outcomes and subgroup definitions are clinically meaningful, while privacy protections should prevent feedback systems from becoming an additional source of surveillance or discrimination. Existing bias-aware clinical frameworks can help institutions connect identified disparities to mitigation duties and organizational accountability [30,31].

Model updates require version-specific assessment. An update may improve average performance while worsening calibration or error for a smaller group. For regulated AI-enabled devices, a predetermined change control plan (PCCP) can prospectively describe planned modifications, methods for development and validation, and impact assessment [32]. Regardless of whether a formal PCCP applies, vendors and institutions should document both the previous and updated versions, affected data and features, subgroup impact, local validation needs, communication duties, and rollback criteria. Drift monitoring may help identify changes in inputs or performance, but a detected shift still requires clinical interpretation and governance action [33].

Explainability can support targeted auditing but should not be treated as proof of fairness or safety. Feature-attribution or visualization methods may help identify reliance on spurious signals, acquisition artifacts, or socially patterned proxies. However, explanations can be unstable, incomplete, or falsely reassuring [34,35]. They should therefore be used alongside provenance review, subgroup performance, calibration, workflow analysis, and outcome monitoring. Table 3 links each lifecycle stage to evidence, authority, a decision gate, and corrective options.

**Table 3:** Lifecycle decision matrix for refined exclusion.

Stage	Minimum evidence	Review and decision authority	Decision gate	Corrective options
Intended use and risk classification	Clinical task, target population, excluded uses, error consequences, and risk tier	Developer and clinical owner; institutional AI governance body approves use	Is the use sufficiently defined and risk appropriate?	Clarify or narrow use, add human review, and do not proceed
Data and development	Composition, event counts, missingness, label and measurement provenance, subgroup learnability, and proxy justification	Developer with clinical and data governance review	Is evidence sufficient to learn and evaluate relevant groups and contexts?	Enrich data, revise target or proxy, restrict population, and seek external evidence
Validation	Discrimination, calibration, threshold errors, clinical utility, and subgroup and intersectional uncertainty	Independent or institutional validation team	Does performance meet the prespecified clinical criteria with acceptable uncertainty?	Proceed, recalibrate, change threshold, conduct additional validation, and stop
Procurement and local adoption	Vendor evidence dossier, regulatory status, known limitations, data access and audit terms, update and monitoring plan, and contractual accountability	Multidisciplinary procurement and AI governance committee	Can the institution safely support the system in its local workflow?	Conditional procurement, staged deployment, negotiate evidence and monitoring duties, and decline
Deployment and monitoring	Model metrics plus overrides, delays, allocation, incidents, and complaints; model and data versioning	Clinical owner and patient safety or AI governance committee with pause authority	Has a prespecified or clinically serious safety signal emerged?	Investigate, add safeguards, restrict, recalibrate, pause, and retire
Updates	Version-specific validation, subgroup impact, and communication and rollback plan	Vendor and institutional change control authority; regulator when applicable	Does the update preserve safety across intended populations and settings?	Approve, require local revalidation, limit update, roll back, and suspend
Patient feedback and incident response	Accessible reporting channel, case linkage, severity, and cluster review	Patient safety office with patient representation and escalation authority	Does feedback indicate a severe event or recurring subgroup or workflow pattern?	Formal audit, targeted subgroup review, workflow redesign, vendor correction, and restriction or suspension

### A Worked Example: Chest Radiograph AI

Consider a hospital evaluating a chest radiograph classifier for urgent abnormality detection. Before purchase, the vendor provides overall and subgroup sensitivity, false-negative rates, calibration where a risk score is used, CIs, site and device composition, intended use limits, and update procedures. The hospital then performs local validation across relevant scanners, care settings, and clinically justified demographic or disease groups. If an intersectional group has too few events for a stable estimate, the committee records an evidence gap rather than inferring safety and may require enhanced radiologist review or restrict autonomous prioritization for that group.

After staged deployment, the hospital monitors false-negative rates, override rates, turnaround times, and diagnostic delays across relevant patient groups and devices. A patient complaint alleging repeated misclassification is linked to the model version and reviewed with comparable cases. A single severe event or a cluster of similar events initiates a formal safety review. The governance committee may continue use with additional safeguards, require recalibration or vendor investigation, narrow the intended use, or pause the system. This example shows how subgroup evidence becomes actionable only when it is connected to workflow data, decision authority, and predefined corrective options.

### Risk-Proportionate Implementation and Practical Constraints

The framework should be proportionate to clinical risk. High-risk diagnostic, triage, treatment recommendation, and care allocation systems require stronger subgroup evidence, local validation, monitoring, and pause authority. Low-risk administrative tools may justify lighter review, although an apparently administrative tool can still affect access or resource allocation. Risk classification should therefore consider not only the model’s stated function but also its actual position in the care pathway and the reversibility of error.

Perfect representation is unattainable, and exhaustive stratification can produce unstable estimates. Institutions should prioritize subgroups based on intended use, plausible biological or social error mechanisms, prior evidence, and the local population. Analyses should report event counts and CIs, distinguish exploratory from prespecified comparisons, and avoid treating race or ethnicity as biological essence. When intersectional analyses are underpowered, hierarchical methods, multisite collaboration, prospective data, and qualitative safety intelligence may supplement but not erase uncertainty.

Sensitive attributes create a genuine tension between equity monitoring, privacy, data minimization, and antidiscrimination law. Refusing to collect any subgroup information can conceal inequity, while uncontrolled collection can create new harms. Governance should specify the lawful purpose, minimum necessary attributes, access controls, retention, analytic methods, and limits on secondary use. Where direct attributes are unavailable or inappropriate, institutions may use clinically justified contextual variables or targeted audits, but proxy inference should not be assumed to reproduce self-identified characteristics accurately.

Resource-constrained institutions may lack sufficient cases, personnel, or infrastructure to perform every function independently. Shared capacity can include multi-institutional validation networks, regulator- or professional society–developed templates, external audits, common incident taxonomies, and vendor-supported monitoring under enforceable contracts. Federated or privacy-preserving multisite evaluation may broaden evidence, and synthetic data may assist testing, but these approaches do not by themselves establish local real-world performance [36,37]. Risk-proportionate minimum standards are preferable to an unfunded mandate that only well-resourced systems can meet.

Responsibility is shared, but authority must be explicit. Developers control data and model design; vendors control documentation, updates, and support; health systems control procurement, workflow integration, and local monitoring; clinicians interpret outputs in care; and regulators establish evidence and postmarket requirements. Shared responsibility should not mean diffused accountability. Every deployment should identify the individual or body authorized to restrict, pause, or retire the system and the conditions under which that authority is exercised.

## Discussion

This viewpoint contributes 4 elements. First, it defines refined exclusion through an evidence deficit affecting a clinically relevant population or context, concealment by aggregate evaluation, concentration of uncertainty or reduced utility, and insufficient governance response. Second, it distinguishes refined exclusion from algorithmic fairness, hidden stratification, dataset shift, subgroup underperformance, and health inequity. Third, it uses data justice to connect representation, procedural traceability, and real-world consequences. Fourth, it translates these principles into lifecycle decision gates with responsible reviewers, corrective options, and explicit pause authority.

The framework complements reporting, audit, and trustworthy AI initiatives rather than creating a competing checklist [7,8,25,27]. Reporting standards make evidence visible, audits examine error pathways, and regulatory and risk management frameworks structure oversight [25,27,29,32]. Refined exclusion adds a more specific institutional question: when aggregate performance coexists with concentrated uncertainty or reduced reliability, who must decide what happens next? Future work should test the proposed indicators, trigger criteria, and response mechanisms across diverse AI applications and health systems.

Several limitations remain. The cases were not selected systematically, and the framework does not estimate the frequency of refined exclusion. Its definition and decision matrix are conceptual and require empirical evaluation. Subgroup categories may be incomplete, unstable, or contested, and downstream harm is often difficult to attribute to a model rather than the surrounding workflow. Governance capacity and legal requirements also vary across jurisdictions. These limitations support a cautious claim: subgroup disparities should not automatically be labeled as harm, but clinically plausible and concentrated disparities should be treated as safety signals rather than obscured by aggregate performance.

### Conclusions

Refined exclusion is distinct from subgroup bias. It describes a governance condition in which aggregate performance masks concentrated uncertainty or reduced clinical reliability, while responsible institutions fail to respond adequately. Data justice makes that condition actionable by linking representation, provenance, validation, monitoring, and patient experience to explicit decision gates. Medical AI should be evaluated not only by its average performance but also by whether its safety can be demonstrated across the populations and settings in which it is used, and whether accountable actors have the authority to restrict or discontinue its use when such safety cannot be established.

## Data Availability

Data sharing is not applicable because no datasets were generated or analyzed for this viewpoint.

## Abbreviations

PCCP: predetermined change control plan
TRIPOD+AI: Transparent Reporting of a Multivariable Prediction Model for Individual Prognosis or Diagnosis–AI
